# P25 When fevers hide secrets: a case report of papillary thyroid carcinoma presenting as secondary haemophagocytic lymphohistiocytosis

**DOI:** 10.1093/rap/rkae117.056

**Published:** 2024-11-05

**Authors:** Jin Ha Kim, Jeyna Irvinn, Luke Sammut

**Affiliations:** Portsmouth Hospitals University NHS Trust, Portsmouth, United Kingdom; Portsmouth Hospitals University NHS Trust, Portsmouth, United Kingdom; Portsmouth Hospitals University NHS Trust, Portsmouth, United Kingdom

## Abstract

**Introduction:**

Haemophagocytic lymphohistiocytosis (HLH) is a rare but potentially life-threatening systemic hyperinflammatory syndrome. HLH can either be primary (resulting from inherited genetic mutations), or secondary (an inappropriate host response to infection, malignancy, or autoimmune disease or other triggers). Primary HLH tends to present in childhood or young adults, whereas secondary HLH presents in adults with a wide range of clinical presentations. We present an uncommon case of a 27-year-old lady with secondary HLH who was initially treated for sepsis and subsequently found to have papillary thyroid cancer.

**Case description:**

The case involved a 27-year-old lady who immigrated from Afghanistan in August 2021, with no further travel history since. As her native language was Pashto, history and clinical updates were completed via her husband and translators. She was initially reviewed in the surgical admissions unit, having presented with sudden-onset abdominal pain and fever. She was discharged the same day following a normal ultrasound scan.

She re-presented three days later with a rash, ongoing fevers, headache, and myalgia, and was admitted to a general medical ward. Observations demonstrated persistent fevers reaching up to 40 degrees Celsius, and treatment for sepsis of unknown source was started with broad-spectrum antibiotics. A full septic screen, extended viral panel, autoimmune screen and imaging were all requested. Chest radiography demonstrated inflammatory changes, though subsequent computed tomography (CT) chest/abdomen/pelvis was normal. Viral serology showed reactivity in Parvovirus B19 IgM and was equivocal for Epstein-Barr virus (EBV).

Her symptoms persisted despite antibiotics and her ferritin levels rose rapidly, peaking at 81820ug/L. An increasing clinical concern for secondary HLH prompted regular speciality reviews by haematology and rheumatology. Bone marrow biopsy confirmed haemophagocytosis, and multiple skin biopsies showed no evidence of clonal T-cell disease. Her case was discussed in the HLH multidisciplinary meeting at UCLH, at which point her H-score was 192 points (80-88% probability of haemophagocytic syndrome). Treatment for secondary HLH was started with intravenous anakinra and methylprednisolone, with good clinical response. Of note, subsequent testing showed a negative Parvovirus B19 DNA PCR and a negative EBV viral load.

PET CT completed upon discharge showed activity in a thyroid nodule. Biopsy later confirmed papillary thyroid carcinoma, which was treated with total thyroidectomy. Both her prednisolone and anakinra have since been carefully withdrawn. She has recovered well and continues under regular follow-up with no recurrence of her HLH.

**Discussion:**

This case highlights the diagnostic complexity and challenges of HLH, and underscores the necessity of maintaining a low threshold of suspicion due to the potential for rapid clinical deterioration and high mortality. Although it is already known that various infections can be associated with secondary HLH, there is a significant overlap of symptoms and laboratory findings between HLH and infections. Our patient also had ‘reactive’ viral studies, which were not consistent with true infection, adding to the diagnostic challenge.

The intense immune activation in HLH can lead to the detection of viral DNA/RNA or antigens in the absence of active infection. This phenomenon is supported by other studies and case reports documenting positive viral tests without evidence of active infection, highlighting the importance of careful interpretation of these results. Understanding this can help avoid unnecessary delays in diagnosis and timely management of HLH.

Early detection of underlying triggers can significantly impact the management and outcome of HLH. In this case, a PET CT revealed activity in a thyroid nodule, leading to a biopsy that confirmed papillary thyroid cancer. Although HLH is known to be associated with solid tumours, papillary thyroid cancer is a rare trigger. This finding emphasises the importance of including neck imaging when investigating secondary causes of HLH.

In summary, this was a case of secondary HLH due to papillary thyroid cancer. This case not only highlights the importance of considering infection as a differential diagnosis in patients with HLH but also emphasises the potential for reactive viral results. Early identification and comprehensive evaluation are crucial for effective management of and improving patient outcomes in this rare but potentially life-threatening condition.

**Key learning points:**

• Early recognition and diagnosis:

• HLH should be considered in patients with persistent fever and systemic symptoms.

• Monitoring markers such as ferritin levels and calculating the H-score can aid in the timely diagnosis of HLH.

• Interpreting viral serology results:

• Positive viral test results in HLH patients should be interpreted with caution, as they may reflect reactive rather than active infections. This is crucial to avoid unnecessary delays in treatment and to focus on managing the hyperinflammatory state.

• Correlation with clinical findings and additional diagnostic markers is essential to differentiate true infections from reactive results.

• Importance of a multidisciplinary approach:

• The involvement of relevant specialties can provide comprehensive care and improve diagnostic accuracy in complex cases of HLH.

• Collaborative efforts among haematology, rheumatology, dermatology and oncology can facilitate prompt and appropriate treatment.

• Consideration of underlying malignancy including thyroid cancer:

• Even in the presence of an apparent infection, underlying malignancy should be considered as a potential trigger for secondary HLH.

• Routine imaging including the neck, as well as PET CT may be beneficial in patients with severe HLH to rule out hidden malignancies or other triggers.

